# Reactive Oxidation Induced Stoichiometric Modulation of Multivalent Vanadium Oxides

**DOI:** 10.1002/smsc.202300171

**Published:** 2024-02-10

**Authors:** Sehwan Song, Dooyong Lee, Yeongjun Son, Yesul Choi, Jiwoong Kim, Seonghoon Han, Jisung Lee, Seokjun Kim, Seung Gyo Jeong, Si‐Heon Lim, Jiafeng Yan, Songkil Kim, Woo Seok Choi, Hyun Ho Kim, Jaeyong Kim, Jong‐Seong Bae, Naoshia Takesue, Chanyong Hwang, Sungkyun Park

**Affiliations:** ^1^ Department of Physics Pusan National University Busan 46241 Korea; ^2^ Quantum Spin Team Korea Research Institute of Standards & Science Daejeon 34113 Korea; ^3^ Department of Physics Education Kyungpook National University Daegu 41566 Korea; ^4^ Center for Scientific Instrumentation Korea Basic Science Institute Daejeon 34133 Korea; ^5^ School of Mechanical Engineering Pusan National University Busan 46241 Korea; ^6^ Department of Physics Sungkyunkwan University Suwon 16419 Korea; ^7^ Department of Energy Engineering Convergence & School of Materials Science and Engineering Kumoh National Institute of Technology Gumi 39177 Korea; ^8^ Department of Physics and Institute for High Pressure Hanyang University Seoul 04763 Korea; ^9^ Busan Center Korea Basic Science Institute Busan 46742 Korea; ^10^ Department of Applied Physics Fukuoka University Fukuoka 814‐0180 Japan; ^11^ Applied Materials Hwaseong 18469 Korea

**Keywords:** multivalent vanadium oxides, phase transitions, reactive oxygen annealing

## Abstract

Vanadium oxides, such as V_2_O_3_, VO_2_, and V_2_O_5_, have attracted considerable attention because of the fascinating physical properties of each oxidation state. On the other hand, precisely controlling the individual oxidation states is difficult due to the sensitivity of oxygen stoichiometry. This article reports that reactive oxygen annealing (ROA) can systematically change the oxidation state of the vanadium oxide films grown on a c‐Al_2_O_3_ substrate compared with typical annealing under O_2_ environments. Chemical, structural, electronic, and electrical analysis confirms the evolution of the vanadium oxide phases from V_2_O_3_ to V_2_O_3_/VO_2_, VO_2_, VO_2_/V_2_O_5_, and V_2_O_5_, showing that the ROA method can control and tune the oxidation state of the highly oxygen‐sensitive multivalent metal oxides.

## Graphical abstract

This study presents reactive oxygen annealing (ROA), a method altering vanadium oxide films' oxidation states efficiently at reduced temperature and time than conventional methods. Analytical validation affirms ROA's efficacy in transitioning the vanadium oxide phase from V_2_O_3_ to V_2_O_5_, offering a novel approach to synthesizing a transition metal oxide films with heightened selectivity for specific oxidation states, culminating in a single phase.

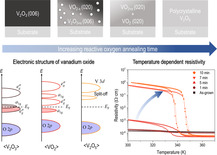

## 1 Introduction

Vanadium oxides (V_2_O_3_, VO_2_, and V_2_O_5_) have attracted considerable attention for the strong correlation effects depending on the oxygen stoichiometry and the corresponding applications. For example, V_2_O_3_ (V^3+^) with a *d* ^2^ system shows the first‐order phase transition from an antiferromagnetic insulator (monoclinic) to a paramagnetic metal (rhombohedral) around 155 K.^[^ ^1^ ^]^ In addition, V_2_O_3_ is a p‐type transparent conducting oxide with potential applications, such as photodetectors and solar cells.^[^ ^2^, ^3^ ^]^ VO_2_ (V^4+^) with a *d* ^1^ system exhibits the first‐order phase transition from an insulating monoclinic phase to a metallic rutile phase around 340 K.^[^ ^4^ ^]^ Therefore, VO_2_ is a promising material for optical switching devices and smart windows owing to the dramatic changes in electrical and optical properties at the phase transition temperature.^[^ ^5^ ^]^ In addition, the complicated phase transition mechanism and various polymorph phases, such as M1, M2, M3, A, and B phases, have prompted considerable research interest in VO_2_.^[^ ^6^, ^7^, ^8^, ^9^ ^]^ Finally, V_2_O_5_ (V^5+^) with a *d* ^0^ system is an insulator with an orthorhombic layered structure.^[^ ^10^ ^]^ The exotic layered structure makes V_2_O_5_ attractive for electrode applications, such as actuators and Li‐ion batteries.^[^ ^8^, ^11^ ^]^ Besides single phases, heterostructures consisting of vanadium oxides have also attracted attention. For example, the VO_2_/V_2_O_5_ heterostructure has the potential to be a photoactive material with superior performance to other materials, including heavy metals.^[^ ^12^ ^]^ On the other hand, realizing and stabilizing individual vanadium oxidation states are still challenging because of the insufficient understanding of the thermodynamic conditions and the sensitivity to oxygen stoichiometry, even though the physical properties of a single oxidation state have been studied extensively.^[^ ^13^, ^14^, ^15^, ^16^, ^17^ ^]^

Many researchers have attempted to grow vanadium oxide films with a single oxidation state using physical vapor deposition methods, such as pulsed laser deposition,^[^ ^6^, ^13^ ^]^ magnetron sputtering,^[^ ^7^, ^18^ ^]^ and molecular beam epitaxy,^[^ ^19^, ^20^ ^]^ resulting in many meaningful results. For example, Lee et al.^[^ ^13^ ^]^ obtained the well‐defined oxidation state of V_2_O_3_, VO_2_, and V_2_O_5_ films using pulsed laser deposition by tuning the oxygen pressure during growth. They also showed that the limited range of oxygen pressure is critical to growing an optimal VO_2_ film, indicating the complication of controlling the oxidation state.

In addition, various posttreatment methods have been proposed to gain oxidation state controllability of a vanadium oxide film. Recently, Lee et al.^[^ ^15^ ^]^ produced different vanadium oxidation states, such as V^3+^(i.e., V_2_O_3_), V^4+^ (i.e., VO_2_), and V^5+^ (i.e., V_2_O_5_) phases, by modifying the oxygen concentrations and substrate temperatures. However, the required substrate temperature (≈1200 °C) is too high, and the reactive time (≈10 h) is also too long in a conventional vacuum system because of the low reactivity of O_2_ gas. Alternatively, O_2_ plasma with higher reactivity has been used to enhance the oxygen reactivity and tune the vanadium oxidation state under ≈500 °C for ≈30 min.^[^ ^21^, ^22^, ^23^ ^]^ Nevertheless, oxygen plasma treatment causes damage to the sample surface due to the direct bombardment of ions. Furthermore, the formation of an unexpected V_2_O_5_ phase by the high reactivity weakens the phase controllability of films. Therefore, a more precise and consistent process for controlling the vanadium oxidation state is needed.^[^ ^24^ ^]^

**Figure 1:**
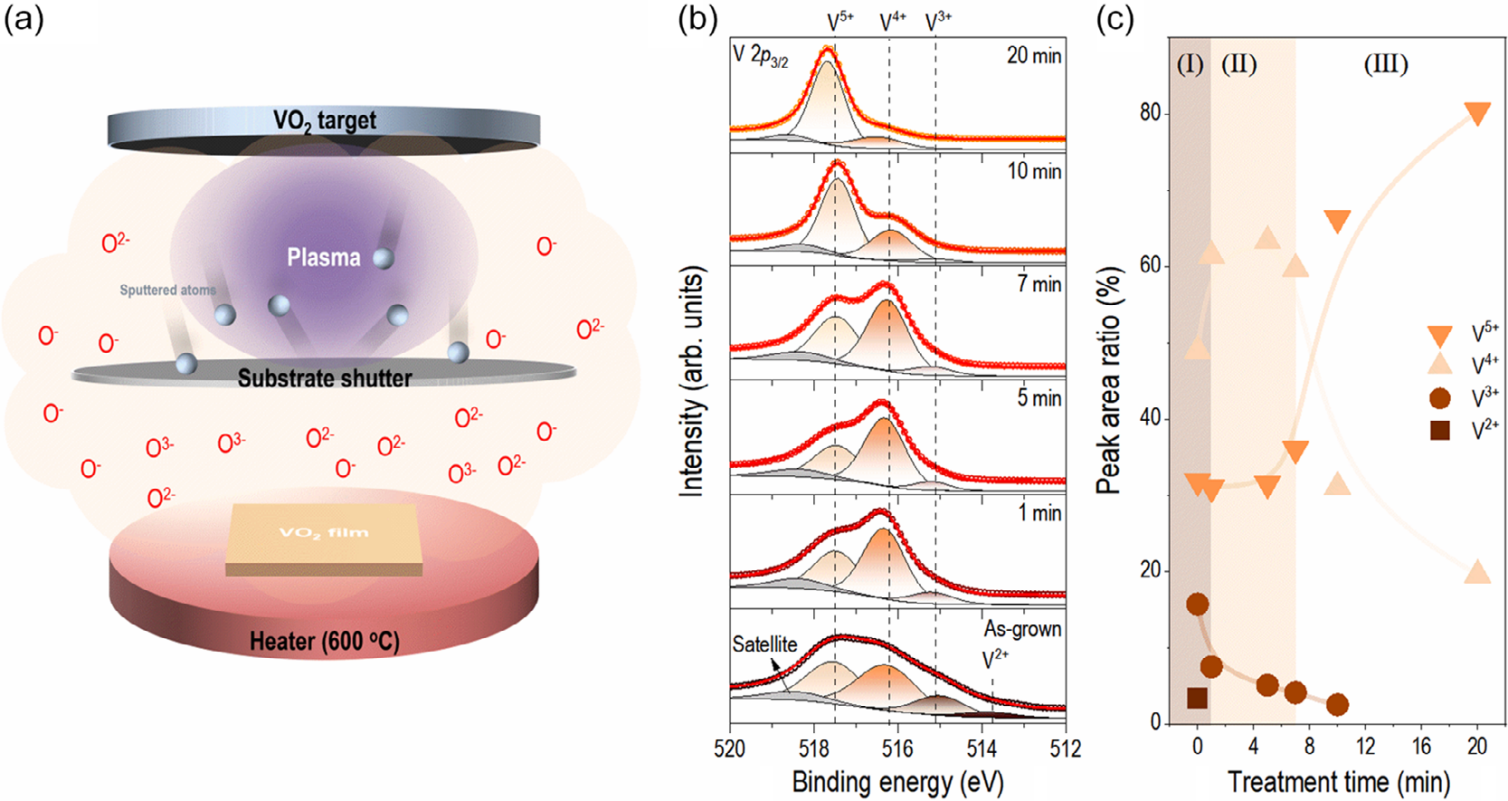
a) Schematic diagram of the ROA method. b) Deconvoluted V 2*p*_3/2_ core‐level XPS spectra of vanadium oxide films with various ROA times. Vertical dashed lines indicate the reference binding energies of V^2+^ (513.7 eV), V^3+^ (515.2 eV), V^4+^ (516.2 eV), and V^5+^ (517.5 eV) in the V 2*p*_3/2_ region.^[^ ^29^, ^30^ ^]^ c) Deconvoluted relative peak areal intensity ratio in the V 2*p*_3/2_ region as a function of the ROA time. The 0 min indicates the as‐grown film.

This study demonstrates the controllability of the oxidation state in the vanadium oxide films grown on a c‐Al_2_O_3_ substrate by reactive oxygen annealing (ROA) generated by the Ar and O_2_ plasma mixture, as shown in **Figure** **1**a. Various oxidation states were obtained by postannealing of as‐grown films for different times under a reactive oxygen gas environment, with the closed shutter (i.e., substrate shutter) to minimize (or avoid) additional deposition and bombardment damage. As a result, a systematic change in the vanadium oxidation state from V^3+^ to V^4+^ to V^5+^ and corresponding structural, electronic, and electrical property variations with increasing ROA time were observed. This approach is advantageous because it is relatively short in time and effective at lower temperatures compared to the previously reported methods. Furthermore, these results demonstrate that reactive oxygen species in oxygen plasma play an important role in effectively changing the oxidation state compared to films annealed in oxygen at the same conditions (e.g., time and temperature).

## 2 Results and Discussion

X‐ray photoelectron spectroscopy (XPS) spectra were taken to investigate the ROA treatment time‐dependent chemical state variation. Before analyzing the spectra, all the spectra were calibrated to the metal oxide peak (530.0 eV) in O 1*s* core‐level XPS spectra (Figure S1a, Supporting Information).^[^ ^25^ ^]^ It was reported that C─C binding energy could be shifted ≈1 eV depending on the electronic band structure or electrical properties.^[^ ^26^ ^]^ On the other hand, V─O bonding in the O 1*s* core‐level XPS spectrum is less sensitive (within 0.1–0.2 eV) depending on the variation of the vanadium valence state.^[^ ^27^, ^28^ ^]^ Figure 1b shows the deconvoluted V 2*p*_3/2_ core‐level XPS spectra of vanadium oxide films with various ROA times. The vertical dashed lines in Figure 1b indicate the reference binding energies of the V^2+^ (513.7 eV), V^3+^ (515.2 eV), V^4+^ (516.2 eV), and V^5+^ (517.5 eV).^[^ ^29^, ^30^ ^]^ V 2*p*_3/2_ regions were fitted with V^2+^, V^3+^, V^4+^, and V^5+^ peaks with V 2*p* satellite (≈518.4 eV). **Table** **1** lists the deconvoluted results, such as the binding energy, full width at half maximum (FWHM), and relative peak areal intensity ratio.

**Table 1:** Binding energy, FWHM, and the relative peak areal intensity ratio of the deconvoluted V 2*p*_3/2_ core‐level XPS spectra of vanadium oxide films with various ROA times

ROA time [min]	Binding energy [eV]				FWHM [eV]				Relative peak areal intensity ratio [%]			
	V^2+^	V^3+^	V^4+^	V^5+^	V^2+^	V^3+^	V^4+^	V^5+^	V^2+^	V^3+^	V^4+^	V^5+^
0	513.8	515.0	516.3	517.5	1.5	1.4	1.6	1.5	3.4	15.6	49.0	32.0
1	–	515.2	516.3	517.5	–	1.2	1.2	1.3	–	7.6	61.4	31.0
5	–	515.2	516.3	517.5	–	1.0	1.1	1.3	–	5.1	63.4	31.5
7	–	515.2	516.2	517.5	–	1.1	1.1	1.2	–	4.1	59.8	36.1
10	–	515.1	516.2	517.4	–	1.2	1.2	1.0	–	2.5	31.1	66.4
20	–	–	516.5	517.6	–	–	1.4	1.0	–	–	20.0	80.0

The relative peak areal intensity ratio of the deconvoluted peaks in the V 2*p*_3/2_ region as a function of the ROA time was calculated to understand the change in the vanadium oxidation state with increasing the ROA time, as shown in Figure 1c. The change in oxidation state can be classified into three regions. 1) The initial V^2+^ and V^3+^ state for the as‐grown film (i.e., 0 min ROA) disappeared and decreased after 1 min ROA. At the same time, the amount of V^4+^ increased while the V^5+^ state remained constant, suggesting that the V^2+^ and V^3+^ were oxidized to V^4+^. 2) There were no significant variations in the vanadium oxidation states from 1 to 7 min. The largest peak area of the V^4+^ state was exhibited, similar to the previously reported epitaxially grown VO_2_.^[^ ^31^, ^32^ ^]^ In contrast, the V^5+^ state peak area ratio remained about ≈30% from 0 to 7 min. This portion of the V^5+^ state was attributed to surface oxidization as the film was exposed to air.^[^ ^29^ ^]^ Generally, Ar‐ion etching can remove the native oxide layer on the surface. However, for multivalent elements, it can cause changes in the valence state of the material, which may cause misinterpretation of the chemical state of the sample.^[^ ^29^ ^]^ 3) Further increases in the ROA time to 20 min resulted in a decrease in V^3+^ and V^4+^ and an increase in V^5+^, indicating that V^3+^ and V^4+^ oxidized to V^5+^. The variation of the relative peak areal intensity ratio as a function of the ROA time shows that the ROA can systematically control the vanadium oxidation state. On the other hand, O_2_ annealed films and all the vanadium V 2*p* spectra exhibit mostly V^5+^ state regardless of the annealing time (Figure S1b, Supporting Information). Furthermore, Figure S2a, Supporting Information, shows the ROA time‐dependent relative atomic ratio of V and O obtained from energy‐dispersive X‐ray spectroscopy (EDS) measurements. The relative atomic ratio of V and O does not change with the ROA treatment time. Also, the V *K*α_1_/O *K*α_1_ ratio along the depth direction, shown in Figure S2b, Supporting Information, remains unchanged. It is worth noting that the change in the amount of oxygen (or V/O ratio) is practically indistinguishable from the EDS measurements due to the limited resolution.

**Figure 2:**
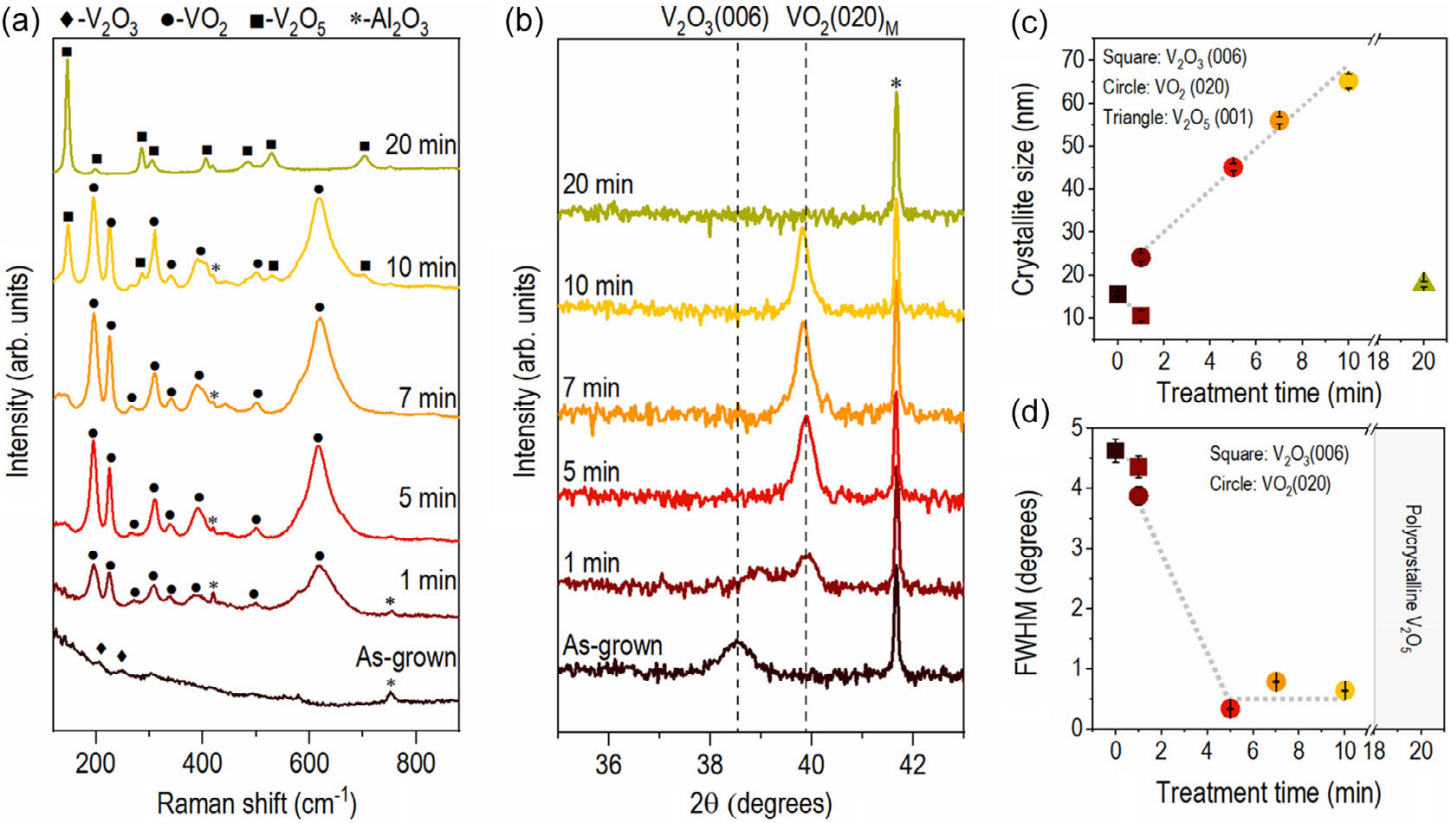
Room‐temperature a) Raman spectra and b) 2*θ*/*ω* XRD patterns of the vanadium oxide films with various ROA treatment times. The dashed lines in (b) indicate the bulk V_2_O_3_(006) peak and monoclinic VO_2_(020) peak position. c) Crystallite size calculated from the XRD patterns and d) FWHM of rocking scan as a function of the ROA treatment time.

To see the chemical state variation depending on ROA time directly reflecting the structural changes of film, the room temperature Raman spectra of the vanadium oxide films treated with various ROA times, shown in **Figure** **2**a, are measured. Raman spectroscopy is used widely to determine the phases of vanadium oxides because distinct Raman modes are exhibited depending on the vanadium oxidation state.^[^ ^23^, ^33^ ^]^ For example, V_2_O_3_ with corundum structure has seven Raman‐active modes (2 *A*_1g_ mode: 240 and 510; 5 *E*_g_ mode: 218, 300, 378, 340, and 600 cm^−1^).^[^ ^33^ ^]^ Monoclinic VO_2_ showed 18 Raman‐active modes (9 *A*_g_: 137, 194, 224, 310, 340, 393, 499, 612, and 633 cm^−1^; 9 *B*_g_: 143, 224, 262, 393, 442, 484, 582, and 820 cm^−1^).^[^ ^34^ ^]^ In particular, Raman peaks at 195 ($\omega_{v1}$), 224 cm^−1^ ($\omega_{v2}$), and 615 cm^−1^ ($\omega_{0}$) are sensitive to the zigzag V─V dimers and V─O bonding.^[^ ^31^, ^35^ ^]^ V_2_O_5_ with an orthorhombic layered structure shows 8 Raman‐active modes (4 *A*_g_: 194, 302, 406, and 526 cm^−1^ and 4 *B*_g_:143, 284, 697, and 707 cm^−1^).^[^ ^23^ ^]^ Among them, the Raman peak around 143 cm^−1^ was derived from the layered structure of V_2_O_5_.^[^ ^23^ ^]^ The as‐grown film showed Raman mode at 752 cm^−1^ (*) derived from the c‐Al_2_O_3_ substrate,^[^^36,37]^ and the weak Raman mode (♦) from a corundum structure V_2_O_3_ at 200 cm^−1^ (*E*_g_ mode) and 250 cm^−1^ (*A*_1g_ mode), as shown in Figure S4, Supporting Information, respectively. The Raman modes of the monoclinic VO_2_ (•) appeared for the film treated for 1 min. They became more assertive and sharper as the ROA time was increased up to 10 min, indicating the formation of the monoclinic VO_2_ phase (Figure S3a, Supporting Information). At 10 min ROA, the Raman modes of the monoclinic VO_2_ (•) and V_2_O_5_ (▪) are observed, suggesting the coexistence of the VO_2_ and V_2_O_5_ phases in the film.^[^ ^36^, ^37^ ^]^ As mentioned above, the Raman mode of ≈195 cm^−1^ (*ω*_v1_) and 224 cm^−1^ (*ω*_v2_) represents the V–V vibration mode of monoclinic VO_2_. Furthermore, the Raman mode of ≈615 cm^−1^ (*ω*_0_) indicates the V–O stretching mode of VO_2_. Specifically, various phases of VO_2_, such as M1, M2, M3, A, and B with different Raman peak positions, can be formed depending on the angle and length of the V–V dimer.^[^ ^38^ ^]^ For example, *ω*_0_ mode varies largely from 615 to 650 cm^−1^ depending on the VO_2_ phase. Figure S3b, Supporting Information, shows the deconvoluted peak position of Raman modes (*ω*_v1_, *ω*_v2_, and *ω*_0_) as a function of ROA time (1–10 min). The peak positions have no noticeable variation, indicating that all films are M1 phase.^[^ ^38^ ^]^ With further increases in ROA time to 20 min, the Raman modes of VO_2_ disappeared while those of V_2_O_5_ only remained. Furthermore, the strong peak at 146.43 cm^−1^ is shown in the Raman spectra for 20 min‐treated film, suggesting the formation of the layered V_2_O_5_ phase. In addition, the homogeneity of the Raman spectra was checked by measuring at four different locations (P1, P2, P3, and P4) on the films. Similar Raman spectra are shown in Figure S4, Supporting Information, for all different locations, suggesting no variation in the sample.

The 2*θ*/*ω* X‐ray diffraction (XRD) patterns of the films were obtained at room temperature to investigate the structural properties of the films, as shown in Figure 2b. All XRD patterns were calibrated to the peak position (2*θ*) of the Al_2_O_3_(006) (41.67°, ICSD #9770). The vertical dashed lines indicate the V_2_O_3_(006) peak of the bulk V_2_O_3_ with corundum structure (38.55°, ICSD #1869) and the VO_2_(020)_M_ peak of monoclinic VO_2_ (39.88°, ICSD #647 604). Only a broader peak was found in the bulk V_2_O_3_(006) peak for the as‐grown film. To determine whether the as‐grown vanadium oxides are V_2_O_3_ or VO_2−*X*_, we measured the *ϕ*‐scan for the as‐grown film, as shown in Figure S5a, Supporting Information. The azimuthal *ϕ*‐scan shows the V_2_O_3_(116) and Al_2_O_3_(116) plane of the as‐grown film. It exhibits sixfold symmetry separated by 60°, confirming V_2_O_3_. Moreover, the peak positions of the substrate and the film match together, suggesting the epitaxial growth of the V_2_O_3_ film on the Al_2_O_3_(0001) substrate.^[^ ^39^ ^]^ On the other hand, the sixfold peaks of monoclinic VO_2_(011) rotated by 30° relative to the Al_2_O_3_(012) peaks must be shown for the VO_2_ film deposited on c‐plane Al_2_O_3_. Furthermore, the sixfold peaks arise from the three equivalent domains of VO_2_ matched with the surface hexagonal plane of c‐plane Al_2_O_3_, indicating the following epitaxial relationship: VO_2_(010)//Al_2_O_3_(0001) and VO_2_[100]//Al_2_O_3_[100].^[^ ^32^ ^]^ However, we could not observe the VO_2_(011) plane associated with data from the *ϕ*‐scan (Figure S5b, Supporting Information). Therefore, the as‐grown film is in the V_2_O_3_ phase, not VO_2−*X*_.

At 1 min ROA, a peak appeared at the bulk VO_2_(020)_M_ position, and the broader V_2_O_3_(006) peak for the as‐grown film shifted to a higher angle. Interstitial oxygen in a V_2_O_3_ phase decreases the *c*‐axis lattice parameter, shifting the V_2_O_3_(006) peak to a higher angle in XRD.^[^ ^40^, ^41^ ^]^ Therefore, the peak at ≈39° (2*θ* value) for the 1 min ROA film was attributed to a V_2_O_3_ phase with excessive oxygen after treatment. The XRD peak related to V_2_O_3_ disappeared, and the intensity of the VO_2_(020) peak increased for the 5 min‐treated films. As the ROA time increased to 10 min, there were no significant changes in the VO_2_(020) peak position, while the FWHM of the peak decreased. For the 20 min‐treated film, the diffraction peaks of V_2_O_3_(006) and VO_2_(020) were not noticed. However, the high energy XRD measurement, shown in Figure S6a, Supporting Information, indicates the presence of polycrystalline V_2_O_5_ peaks even though the relative peak intensity is small. Furthermore, the in‐plane XRD measurement in the wide range (10–90°) reveals the polycrystalline V_2_O_5_ phase clearly (Figure S6b, Supporting Information). Figure 2c,d shows the crystallite size and the FWHM of the rocking curves of the films. The crystallite size (*D*) is obtained from the Scherrer equation $D=\frac{K\lambda }{\beta \cos\theta }$, where *K* is the shape factor (0.9), *β* is the FWHM, and *θ* is the peak position in radian.^[^ ^42^ ^]^ Also, the crystallite size of the monoclinic VO_2_ phase (circle) increased with the ROA time to 10 min. However, for the 20 min‐treated film, small crystallinity from the polycrystalline V_2_O_5_ phase was observed. **Table** **2** lists the crystallite size of the vanadium oxide films. The FWHM of the rocking curve, related to the VO_2_ phase (i.e., (020) peak), sharply decreased between 1 and 5 min and was relatively constant after 5–10 min. In particular, the FWHM for 5 min ROA film was 0.34°, smaller than that of the optimized VO_2_ film grown on an Al_2_O_3_ substrate by RF‐magnetron sputtering.^[^ ^31^, ^43^ ^]^ Figure S7, Supporting Information, compares 2*θ*/*ω* XRD patterns between postannealed film under O_2_ gas at 600 °C and ROA‐treated films at various times (0, 1, 3, 7, 10, and 20 min). Unlike the ROA‐treated films, the film annealed under O_2_ gas (Figure S7, Supporting Information) showed only the V_2_O_3_(006) peak regardless of the annealing time, similar to the as‐grown film, even though the peak position and FWHM are slightly different depending on the annealing time due to the thermal effect. These results indicate that the ROA processes chemically and structurally transformed V_2_O_3_ to monoclinic VO_2_, then to the mixture of monoclinic VO_2_ and layered V_2_O_5_, and finally to layered V_2_O_5_ as the treatment time increased.

**Table 2:** Crystallite size calculated from the XRD patterns by the Scherrer equation

ROA time [min]	V_2_O_3_(006)	VO_2_(020)	V_2_O_5_(001)
0	15.6 ± 0.47 nm	–	–
1	10.5 ± 1.17 nm	24.1 ± 1.06 nm	–
5	–	45.1 ± 0.87 nm	–
7	–	56.0 ± 0.89 nm	–
10	–	65.1 ± 1.59 nm	–
20	–	–	17.9 ± 0.68 nm

**Figure 3:**
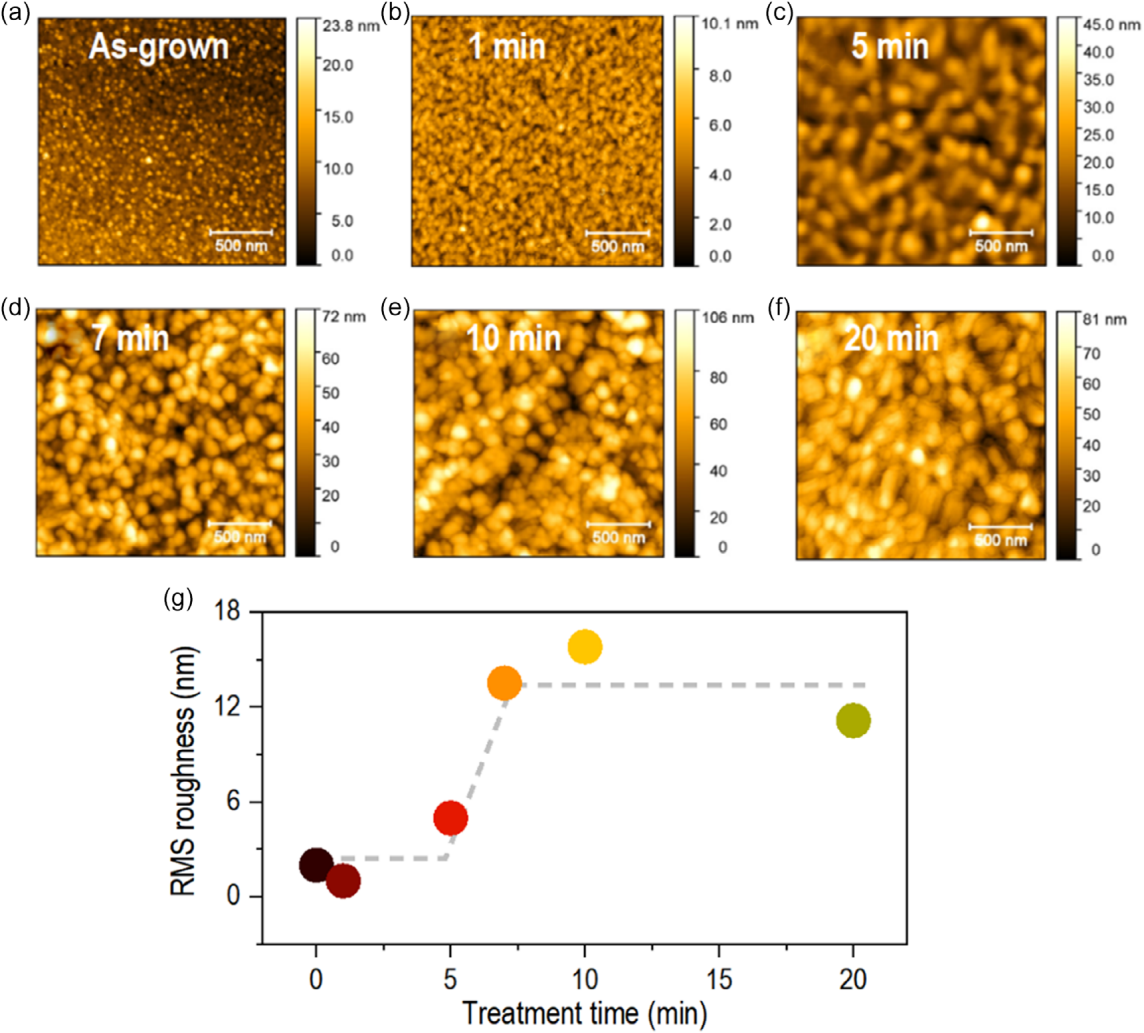
a–f) AFM images of the vanadium oxide films with increasing ROA time. g) RMS roughness of the vanadium oxide films as a function of the ROA time.

The surface morphology variation during ROA is another important aspect for utilizing multivalent oxide‐based heterostructure for various applications. **Figure** **3**a–f shows the surface topography of vanadium oxide films with various ROA times to examine the damage during ROA. At 0 min (i.e., as‐grown film), the film surface exhibited a smaller grain size (Figure 3a). The average vertical grain size (Figure S8b, Supporting Information) calculated from the line profile (Figure S8a, Supporting Information) increased at 5 min and remained almost constant to ≈30 nm as the ROA time increased further. Similarly, the surface root‐mean‐square (RMS) roughness increased after 5 min and was relatively constant after 7 min ROA (Figure 3g). The rapid increase in RMS roughness between 5 and 7 min was attributed to the aggregation of the small grain into a large grain, as evident in the atomic force microscopy (AFM) images (Figure 3c,d). The increased grain size and the RMS roughness can be understood not by surface damage but by reconstructing the microstructure on the film surface because of the highly reactive oxygen ions.^[^ ^22^ ^]^ High‐reactive oxygen delivers sufficient energy to the film surface to cause the surface grain to agglomerate and change shape, resulting in a large grain size, as shown in Figure 3c–f. In addition, the addition deposition effect (i.e., thickness variation) is also examined. Figure S9, Supporting Information, shows a cross‐sectional field‐emission scanning electron microscope (FE‐SEM) image of the vanadium oxide films with increasing ROA time. The roughness of the surface and grain size seems to increase due to the reconstruction of the microstructure, similar to the AFM results shown in Figure 3. Furthermore, the thickness slightly increases with the increasing ROA time. Notably, the bulk density of various vanadium oxides is different. For example, V_2_O_3_ is 4.87 g cm^−3^, VO_2_ is 4.57 g cm^−3^, and V_2_O_5_ is 3.36 g cm^−3^. Therefore, increased film thickness with increased ROA time is attributed not to additional deposition but to reduced film density.

**Figure 4:**
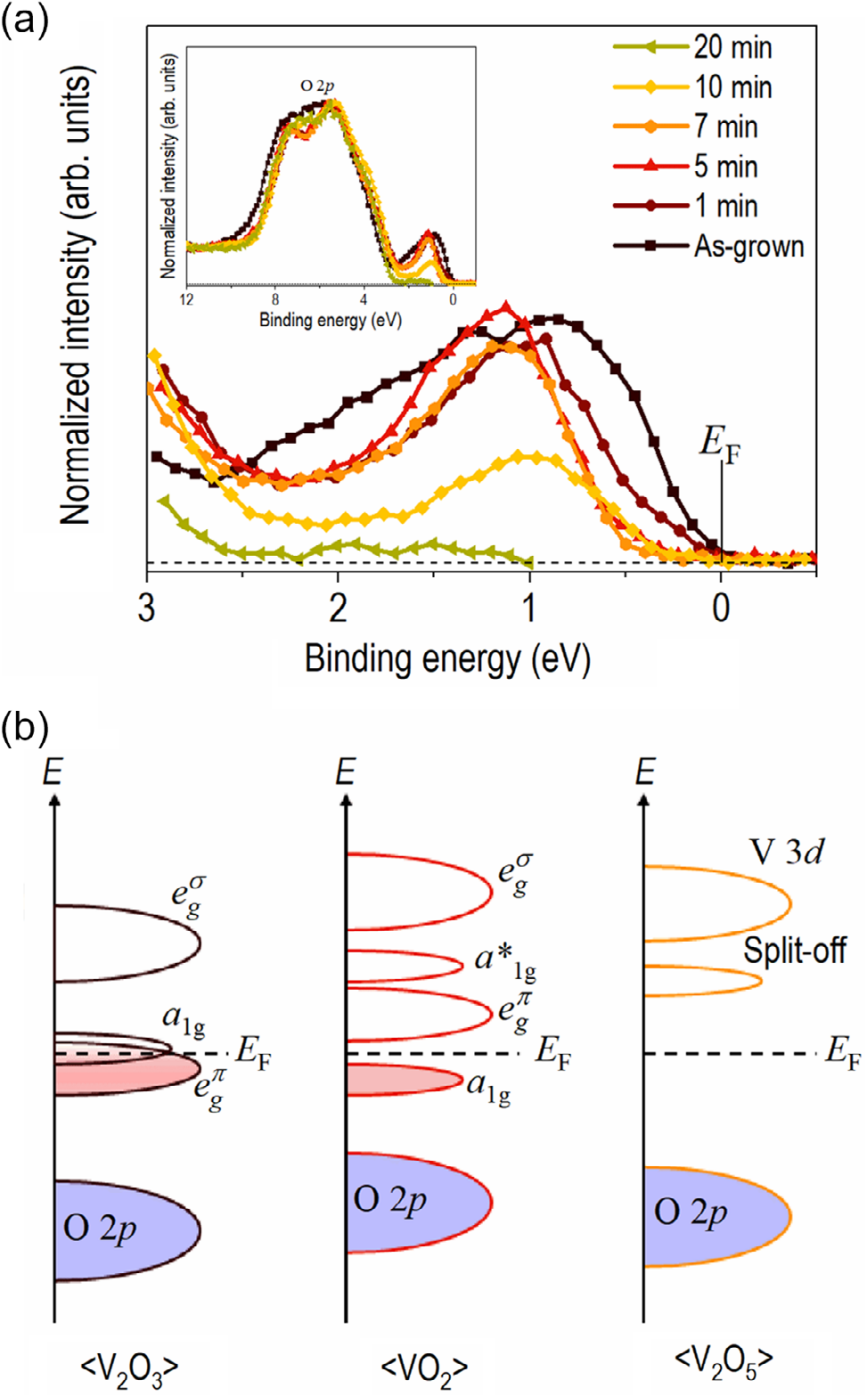
a) VB spectra of the vanadium oxide films with various ROA times and b) energy‐level schematics of bulk V_2_O_3_,^[^ ^39^, ^40^ ^]^ VO_2_,^[^ ^27^ ^]^ and V_2_O_5_^[^ ^41^ ^]^ for comparison. The inset shows the expanded region of VB. Vertical solid‐line indicates the Fermi level (*E*_F_, binding energy = 0 eV) and horizontal dotted‐line indicates the baseline (intensity = 0).

The electronic structure variation depending on ROA time is also examined by measuring valence band (VB) spectra and plotting the energy‐level schematics of bulk V_2_O_3_, VO_2_, and V_2_O_5_, as shown in **Figure** **4**. All films have the O 2*p* band between ≈2 and ≈10 eV below the Fermi level (*E*_F_) because of V 3*d*–O 2*p* hybridization, regardless of the oxidation state, as shown in the inset of Figure 4a.^[^ ^44^ ^]^ The VB spectrum near the *E*_F_ showed significant differences because of the difference in the number of *d‐*electrons and the crystal structure depending on the vanadium oxidation state. The valence band maximum (VBM) corresponding to the disappearance of the spectral weight was obtained by extrapolating the VB spectra (Figure S10, Supporting Information), and the energies of the VBM are listed in Table S3, Supporting Information. In the case of as‐grown film, the strong spectral weight was observed near the *E*_F_, which is consistent with the known electronic structure of V_2_O_3_ consisting of two *d* electrons distributed in the $e_{g}^{\pi }$ and *a*_1g_ bands (Figure 4b).^[^ ^40^, ^45^ ^]^ For the 1 min‐treated film, the spectral weight decreased (not disappeared) near the *E*_F_, suggesting that the amount of the V_2_O_3_ (VO_2_) phase decreased (increased). This is consistent with the XRD results. The spectral weight near the *E*_F_ disappeared as the ROA time was increased to 5 and 7 min. In addition, the VBM shifted from 0.24 ± 0.05 eV for 1 min ROA to a higher binding energy of 0.64 ± 0.06 eV for 5 min ROA and 0.68 ± 0.07 eV for 7 min ROA. This was attributed to a lower bonding *a*_1g_ band of monoclinic VO_2_. For the bulk monoclinic VO_2_, the *a*_1g_ band split into a lower bonding *a*_1g_ (lower Hubbard band) filled one *d* electron and an upper antibonding a*_1g_ and (upper Hubbard band) because of the Peierls instability or Mott–Hubbard transition at room temperature, as shown in Figure 4b.^[^ ^27^ ^]^ For the 10 min‐treated film, although the position of the VBM shifted to lower energy compared to 5 and 7 min, the spectral weight decreased significantly, indicating a decrease in the *a*_1g_ (lower Hubbard band) portion of the VO_2_ phase. Finally, the spectra weight near the *E*_F_ in the 20 min‐treated film disappeared near the *E*_F_. In the case of the orthorhombic V_2_O_5_, an intermediate V 3*d* band was separated from the original V 3*d* band, forming a “split‐off” band below the original V 3*d* band not near *E*_F_, as shown in Figure 4b. No band was filled with electrons near the *E*_F_ because of this characteristic. Therefore, the VB spectra of the 20 min‐treated film are the characteristics of the electronic structure of V_2_O_5_.^[^ ^46^ ^]^ However, there is still a little spectral weight near 1.8 eV, attributed to a residual VO_2_ phase in the majority V_2_O_5_ phase. Interestingly, there is no sign of electrical phase transition characteristics of the VO_2_ phase (i.e., insulator–metal transition [IMT]) despite the remaining partial phase in the valence spectrum for the 20 min‐treated film. It is worth noting that the resistance (not shown) was too large to be measured for the 20 min‐treated film.

The optical conductivity (*σ*_1_) spectra of the films at room temperature are needed to understand the electronic structure better (Figure S11, Supporting Information). A feature of the Drude contribution was exhibited for the as‐grown and the 1 min‐treated film owing to the metallicity.^[^ ^45^, ^47^ ^]^ The Drude conductivity feature disappeared as the ROA time was increased to 10 min, and a new spectra weight of ≈0.8 eV appeared. The position of the spectra weight was similar to the energy of the optical transition from the *a*_1g_ peak to the $e_{g}^{\pi }$ peak in the VO_2_.^[^ ^45^ ^]^ For the 20 min‐treated film, the spectra weight moved to a higher photon energy (≈1.3 eV), which may be due to the optical transition energy (≈1.95 eV) from the O 2*p* band to the “split‐off” band.^[^ ^46^ ^]^ Therefore, the evolution of the real part of the optical conductivity is consistent with that of the VB spectra.

**Figure 5:**
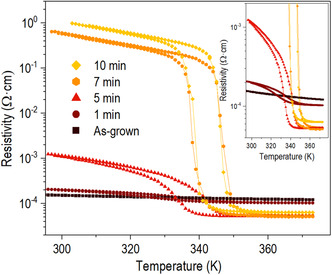
Temperature‐dependent resistivity of vanadium oxide films grown on c‐Al_2_O_3_ with various ROA times. The inset indicates enlarger for as‐grown, 1 min‐, and 5 min‐treated films.

Finally, the temperature‐dependent (from 300 to 370 K) electrical resistivity (*ρ*) of the vanadium oxide films with various ROA times using in‐line fore probes is shown in **Figure** **5**. As expected no phase transition characteristics were noted near the room temperature for the as‐grown film. On the other hand, the broader IMT, which is higher than that of the bulk V_2_O_3_ (155 K), was observed (Figure S12a, Supporting Information). This may be attributed to the excess oxygen and disorder‐induced nonstoichiometry in the film.^[^ ^48^ ^]^ For the 1 min‐treated film, two distinct phase transitions because of the coexistence of V_2_O_3_ and VO_2_ were observed at 203.1 and 326.8 K, respectively (Figure 5 and S12b, Supporting Information). As the ROA time increased, all films except for 20 min‐treated film showed the IMT characteristics near room temperature despite the difference in the transition temperature and resistivity ratios. For the 20 min‐treated film, the resistance (not shown) was too large to be measured. This is consistent with the electrical characteristics of V_2_O_5_.^[^ ^17^ ^]^ In particular, the resistivity of the as‐grown and the 1 min‐treated film above 355 K was higher than that of other films because of the smaller grain size (Figure 3) (i.e., higher density of grain boundaries), including a disordered atomic structure.^[^ ^49^ ^]^ **Table** **3** lists the detailed IMT characteristics, such as average transition temperature, sharpness, hysteresis width, and resistivity change ratio. Despite showing similar structural and chemical properties, the 5 min‐treated film exhibited a lower IMT temperature and resistivity ratio than the 7 min‐treated film. The suppressed IMT characteristics for the 5 min‐treated film might be related to a point defect that is difficult to measure. The resistivity change ratio and sharpness of IMT increased for the 7 and 10 min‐treated film and were similar to bulk VO_2_ characteristics. On the other hand, the film annealed with O_2_ shows a slight decrease in the resistivity with increasing annealing time. Still, no phase transition characteristic is observed due to the semiconductor characteristics of V_2_O_3_ (Figure S13, Supporting Information). Therefore, the ROA treatment controls the systematic transformation from V_2_O_3_ to V_2_O_5_ based on the temperature‐dependent electrical resistivity of the film.

**Table 3:** IMT characteristics, such as the average IMT temperature ($T_{C}^{\text{ave}}$), hysteresis width (Δ*T*), sharpness (Δ*H*) during heating and cooling, and resistivity changes ratio (*ρ*_300 K_/*ρ*_370 K_) of vanadium oxide films for various ROA times

ROA time [min]	$T_{C}^{\text{ave}}$ [K]	Δ*T* [K]	Δ*H* [K]		*ρ*_300 K_/*ρ*_370 K_
			Heating	Cooling	
0	206.5	12.5	46.4	52.9	1.24
1	212.2	7.1	28.5	32.2	1.91
	329.4	20.7	18.9	20.1	
5	332.9	19.1	9.7	10.3	2.00 × 10^1^
7	343.1	14.9	3.4	3.6	1.02 × 10^4^
10	341.4	9.4	3.2	3.3	1.38 × 10^4^

## 3 Summary

This study reported the advantageous ROA method for the evolution of vanadium oxide films in relatively short times and at low temperatures by adjusting reaction time compared with the O_2_ annealing environment. As the various reactive oxygen species could enter the defect and/or interstitial site of the film and combine with vanadium ions, forming various oxidation states, the ROA makes it easier to control the oxidation/chemical state than postannealing in O_2_. By systematic analysis, we demonstrated that the different chemical states of vanadium oxide could be achieved by tuning ROA time. Specifically, VO_2_ and V_2_O_5_ single‐phase films could be obtained at 7 and 20 min, respectively. Notably, the 7 min ROA‐treated film exhibited structurally and electrically similar properties to bulk VO_2_, which is difficult to stabilize because of its narrow growth window. Therefore, this approach provides a novel method for synthesizing a transition metal oxide film with high sensitivity to the oxidation state into a single phase.

## 4 Experimental Section

Vanadium oxide films were grown on an Al_2_O_3_(0001) substrate by RF‐magnetron sputtering from a VO_2_ target (99.9%, Kurt J. Lesker). The base pressure of the sputtering chamber before the deposition was 5.0 × 10^−7^ Torr. During growth, the vacuum chamber was kept at 2 mTorr by flowing the Ar (5 N) gas (i.e., 10 sccm), substrate temperature maintained at 600 °C, and deposition power was 80 W for 10 min. After growth, the films were annealed various times (1, 5, 7, 10, and 20 min) under a gas flow of Ar 9 sccm and O_2_ 1 sccm, with the closed substrate shutter. Furthermore, as‐grown films were annealed under an O_2_ environment at the same conditions (time and temperature) to compare the effect of ROA.

Ex‐situ XPS (AXIS SUPRA, Kratos) with Al *K*α radiation (1486.69 eV), a pass energy of 20 eV, and a step energy of 0.1 eV was performed to examine the chemical state of vanadium oxide films at room temperature. Also, the charging effect for the insulating samples was removed using low‐energy electrons created by the charge‐neutralizer gun with 0.4 A for filament current, 1.0 V for bias voltage, and 4.7 V for charge balance voltage.^[^ ^24^ ^]^ The thickness and atomic ratio of vanadium oxide films were studied using a FE‐SEM (GEMINI500, ZEISS) and EDS (X‐Max 50, Oxford Instruments), respectively. The structural properties of vanadium oxide films were measured by Raman spectroscopy (inVia Raman Microscope, Renishaw) with 50× objective using 532 nm laser and high‐resolution XRD with Cu *K*α, *λ* = 1.5406 Å (Empyrean, PANalytical and SmartLab, Rigaku). The surface morphology was examined by AFM (XE7, Park Systems) with noncontact mode to minimize surface damage. The room temperature optical conductivity (*σ*_1_) of vanadium oxide films was investigated using spectroscopic ellipsometry (M‐2000 and IR‐VASE MARK‐2, J. A. Woollam Co.). The optical spectra were observed between 0.2 and 1.8 eV for incident angles of 55°, 60°, and 65°. Temperature‐dependent resistivity measurement was carried out as in‐line four probes in high temperature (300–400 K) and the van der Pauw method in low temperature (50–300 K) in vacuum around 10^−3^ Torr using Keithley 2400 multimeter, respectively. The specific parameters, probing region, and probing sensitivity of the experimental tools used in this work are listed in Table S1 and S2, Supporting Information, respectively.

## Conflict of Interest

The authors declare no conflict of interest.

## Supporting information

Supplementary Material

## Data Availability

Research data are not shared.
