# Supplementary material for: Reactive Oxidation Induced Stoichiometric Modulation of Multivalent Vanadium Oxides
Source: Small Sci. 2024 Feb 10;4(4):2300171. doi: 10.1002/smsc.202300171 (PMC11935193; doi:10.1002/smsc.202300171)
Supplement: Supplementary file 1 — Supplementary Material [file SMSC-4-2300171-s001.pdf]

Reactive Oxidation Induced Stoichiometric Modulation of Multivalent Vanadium Oxides

Sehwan Song[1,10,*], Dooyong Lee[1,2,*], Yeongjun Son[1,*], Yesul Choi[1], Jiwoong Kim[1,†], Seunghoon Han[1], Jisung Lee[1,3], Seokjun Kim[4], Seung Gyo Jeong[5], Si-Heon Lim[6], Yan Jiafeng[7], Songkil Kim[4], Woo Seok Choi[5], Hyun-Ho Kim[6], Jaeyong Kim[7], Jong-Seong Bae[8], Naoshia Takesue[9], Chanyong Hwang[10] and Sungkyun Park[1,‡]

[1]*Department of Physics, Pusan National University, Busan 46241, Korea*
[2]*Department of Physics Education, Kyungpook National University, Daegu 41566, Korea*
[3]*Center for Scientific Instrumentation, Korea Basic Science Institute, Daejeon 34133, Korea*
[4]*School of Mechanical Engineering, Pusan National University, Busan 46241, Korea*
[5]*Department of Physics, Sungkyunkwan University, Suwon 16419, Korea*
[6]*Department of Energy Engineering Convergence & School of Materials Science and Engineering, Kumoh National Institute of Technology, Gumi 39177, Korea*
[7]*Department of Physics and Institute for High Pressure, Hanyang University, Seoul 04763, Korea*
[8]*Busan Center, Korea Basic Science Institute, Busan 46742, Korea*
[9]*Dearptement of Applied Physics, Fukuoka University, Fukuoka 814-0180, Japan*
[10]*Quantum Spin Team, Korea Research Institute of Standards & Science, Daejeon 34113, Korea*

[*] All of them are equally contributed.

[†] Current address: Applied Materials, Hwaseong 18469, Korea.

[‡] Correspondence should be addressed to S. Park (psk@pusan.ac.kr).

Table S1. Specific conditions for each experiment.

<table>
<tr><th>Experimental methods</th><th colspan="3">Specific parameters used in this work</th><th>Environment</th></tr>
<tr><td rowspan="2">RF-magnetron sputtering</td><td>RF power</td><td>Temperature</td><td>ROA time</td><td rowspan="2">Vacuum</td></tr>
<tr><td>80 W</td><td>600 °C</td><td>1, 5, 7, 10, 20 min</td></tr>
<tr><td rowspan="2">Temperature-dependent resistivity</td><td>Temperature</td><td>Current</td><td>Geometry</td><td rowspan="2">Vacuum</td></tr>
<tr><td>300 ~ 380 K (High temperature)
50 ~ 300 K (Low temperature)</td><td>10 μA</td><td>In-line four-point probe (High temperature)
Van der Pauw (Low temperature)</td></tr>
<tr><td rowspan="2">XPS</td><td>X-ray energy</td><td>Charge neutralizer gun</td><td>Scan step</td><td rowspan="2">Vacuum</td></tr>
<tr><td>1486.69 eV</td><td>Filament current: 0.4 A
Bias voltage: 1.0 V
Charge baleen voltage: 4.7 V</td><td>0.1 eV</td></tr>
<tr><td rowspan="2">Raman</td><td>Laser wavelength</td><td colspan="2" rowspan="2">-</td><td rowspan="2">Ambient</td></tr>
<tr><td>532 nm</td></tr>
<tr><td rowspan="2">XRD</td><td>X-ray source</td><td colspan="2">Power</td><td rowspan="2">Ambient</td></tr>
<tr><td>Cu Kα, λ = 1.5406 Å</td><td colspan="2">40 kV, 40 mA</td></tr>
<tr><td rowspan="2">AFM</td><td>Scan mode</td><td colspan="2">Scan area</td><td rowspan="2">Ambient</td></tr>
<tr><td>Non-contact mode</td><td colspan="2">1.5 μm x 1.5 μm</td></tr>
<tr><td rowspan="2">Ellipsometry</td><td>Incident angle</td><td colspan="2">Scan range</td><td rowspan="2">Ambient</td></tr>
<tr><td>55, 60, 65°</td><td colspan="2">0.2 ~ 1.8 eV</td></tr>
<tr><td rowspan="2">FE-SEM+EDS</td><td>Accelerating voltage</td><td colspan="2">Magnification</td><td rowspan="2">Vacuum</td></tr>
<tr><td>15 keV</td><td colspan="2">x 100 K</td></tr>
</table>

Table S2. Probing region and sensitivity of the experimental tools used in this work.

Methods	Probe depth	Sensitivity
XPS	Sub-nanometer	Surface sensitive
AFM	Sub angstrom	Surface sensitive
Raman	sub-micrometer	Surface and bulk-sensitive
XRD	Hundreds of micrometers	Surface and bulk-sensitive
Temperature-dependent resistivity	Sub nm to millimeter depends on the materials	Surface and bulk-sensitive
FE-SEM and EDS	Hundreds of micrometers	Surface and bulk-sensitive
Ellipsometry (Optical conductivity)	Hundreds of micrometers	Surface and bulk-sensitive

Table S3. Valence band maximum energy obtained from the extrapolation of valence band spectra.

ROA time (min)	Valence band maximum (eV)
0	0.07 ± 0.01
1	0.24 ± 0.05
5	0.64 ± 0.06
7	0.68 ± 0.07
10	0.35 ± 0.11
20	0.94 ± 0.24

Table S4. ROA time-dependent dominant phases of vanadium oxide film for various measurements.

ROA time (min)	XPS	Raman	XRD	Temperature-dependent resistivity	Ellipsometry
0	V_2O_3	V_2O_3	V_2O_3	V_2O_3	V_2O_3
1	VO_2	VO_2	V_2O_3+VO_2	VO_2+VO_2	V_2O_3+VO_2
5	VO_2	VO_2	VO_2	VO_2	VO_2
7	VO_2	VO_2	VO_2	VO_2	VO_2
10	V_2O_5	VO_2	VO_2	VO_2	VO_2
20	V_2O_5	V_2O_5	V_2O_5	V_2O_5	V_2O_5

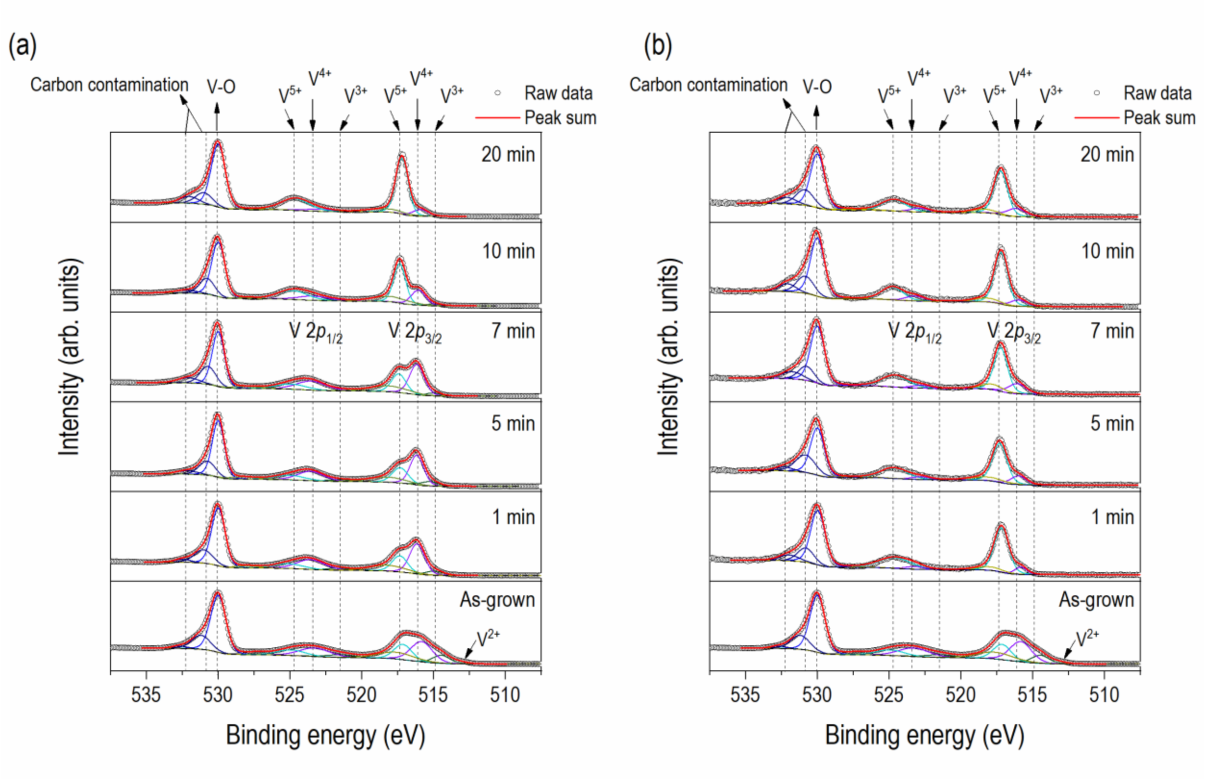

Figure S1. Deconvoluted O 1*s* and V 2*p* core-level X-ray photoelectron spectra of vanadium oxide films with various (a) ROA times and (b) O_2 gas-annealing. Vertical dashed lines indicate the reference binding energies.[26, 27]

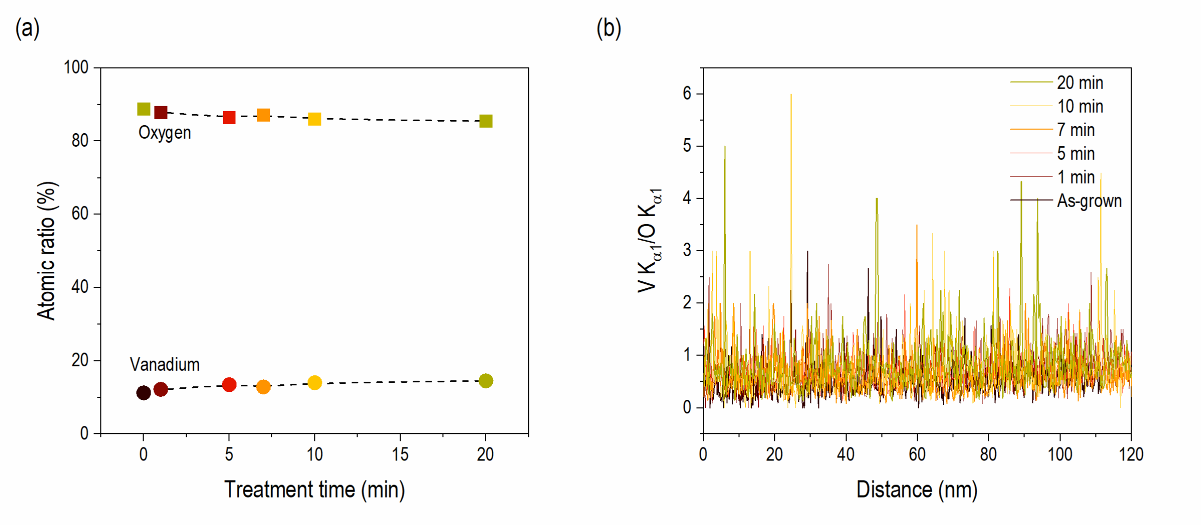

Figure S2. ROA time dependent (a) relative atomic ratio of oxygen and vanadium and (b) intensity ratio of V $K_{\alpha 1}$/O $K_{\alpha 1}$ along the film-depth direction from EDS measurement.

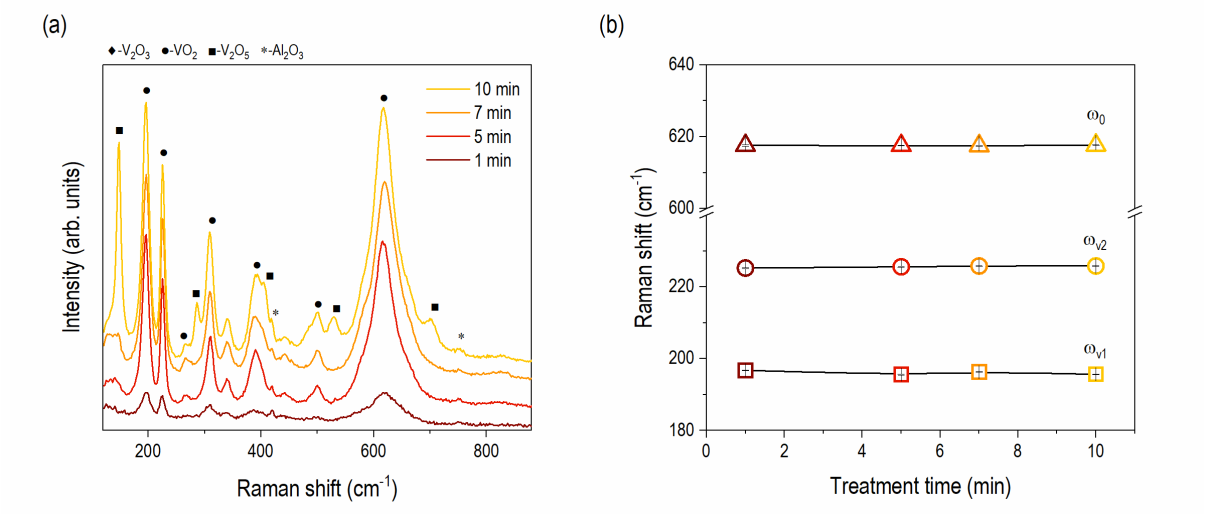

Figure S3. (a) Comparison of Raman spectra for 1-, 5-, 7-, and 10-min film and peak position of ω_{v1}, ω_{v2} and ω_0 depending on the ROA time.

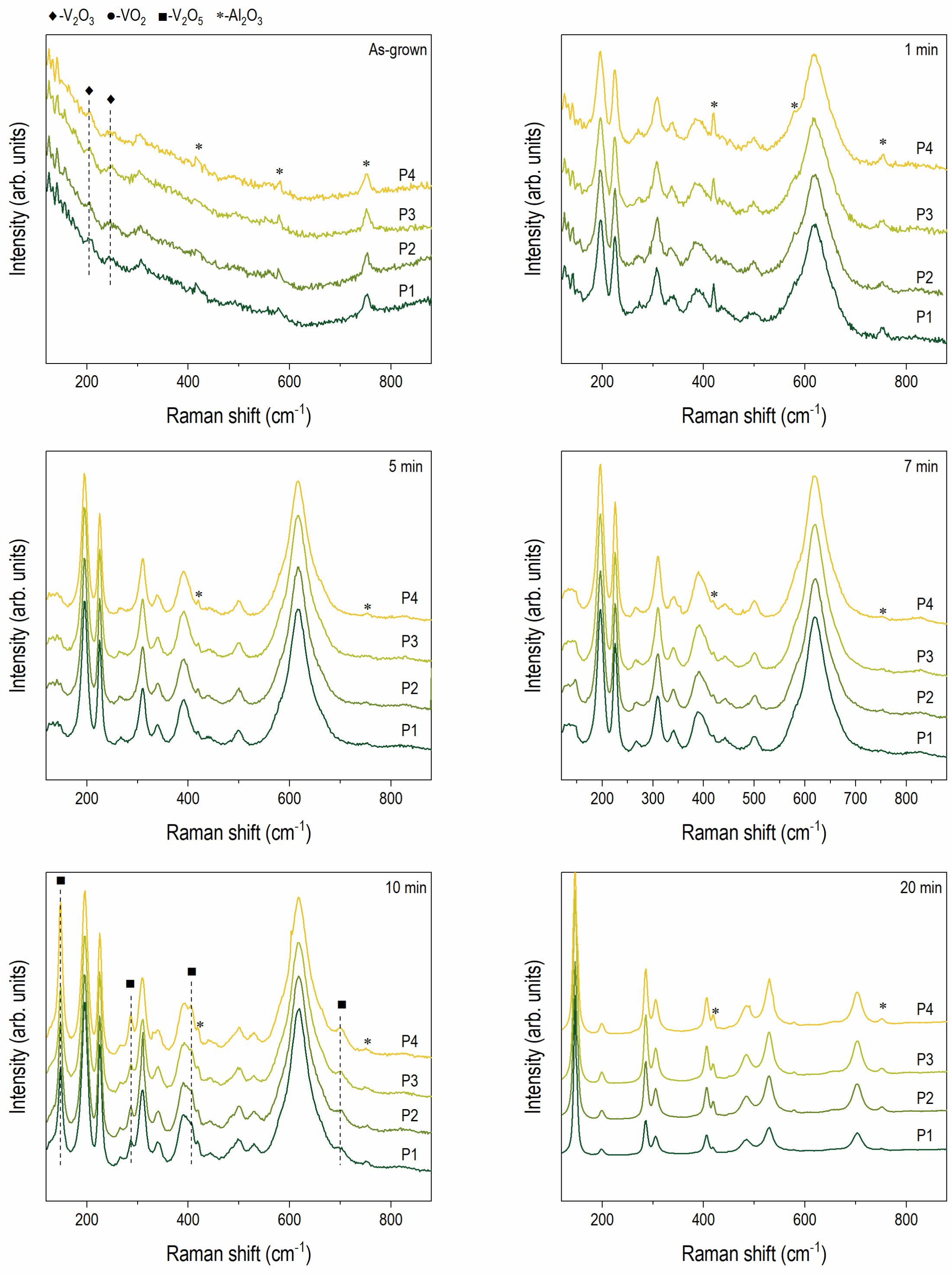

Figure S4. Raman spectra at four different positions in the film with various ROA treatment times.

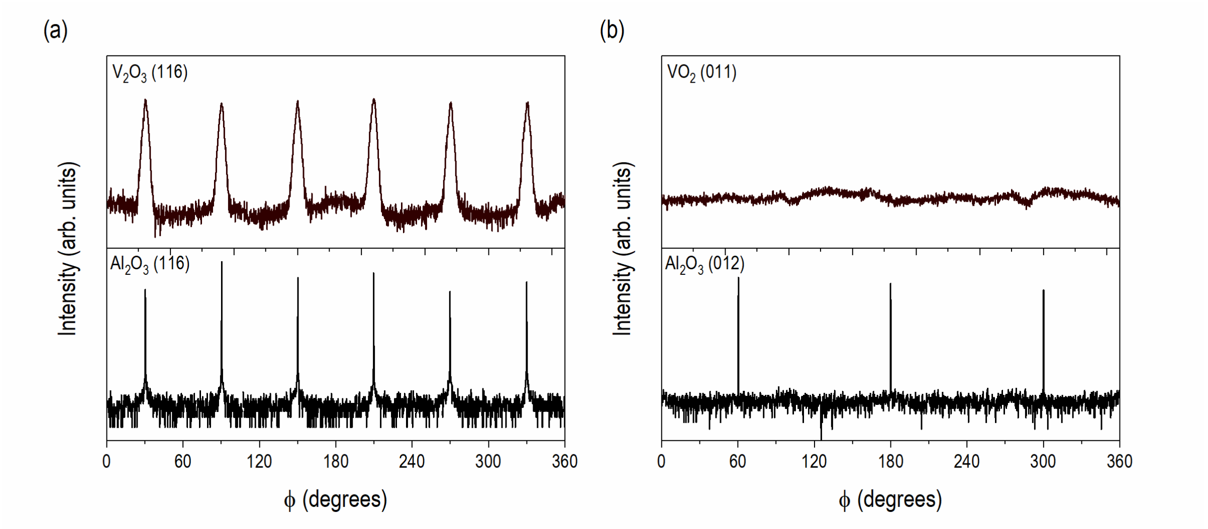

Figure S5. XRD ϕ-scan of (a) V_2O_3(116), Al_2O_3(116) and (b) VO_2(011), Al_2O_3(012) plane for as-grown film.

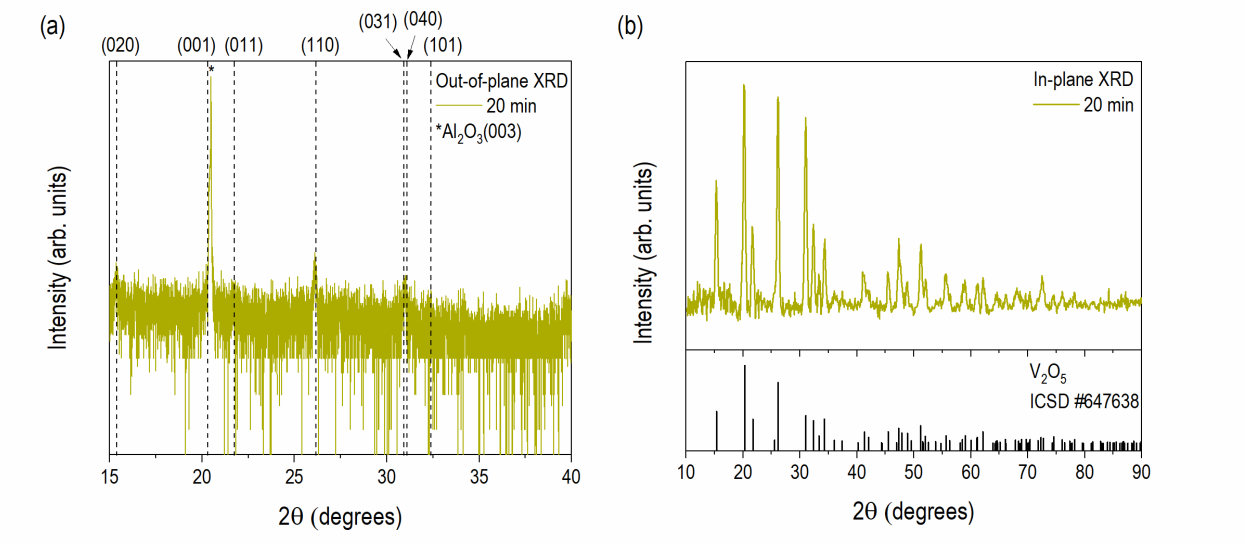

Figure S6. Room temperature (a) out-of-plane and (b) in-plane XRD of 20 min-treated film. Vertical dashed lines and the right bottom figure indicate the reference pattern of bulk V_2O_5 (ICSD- #647638).

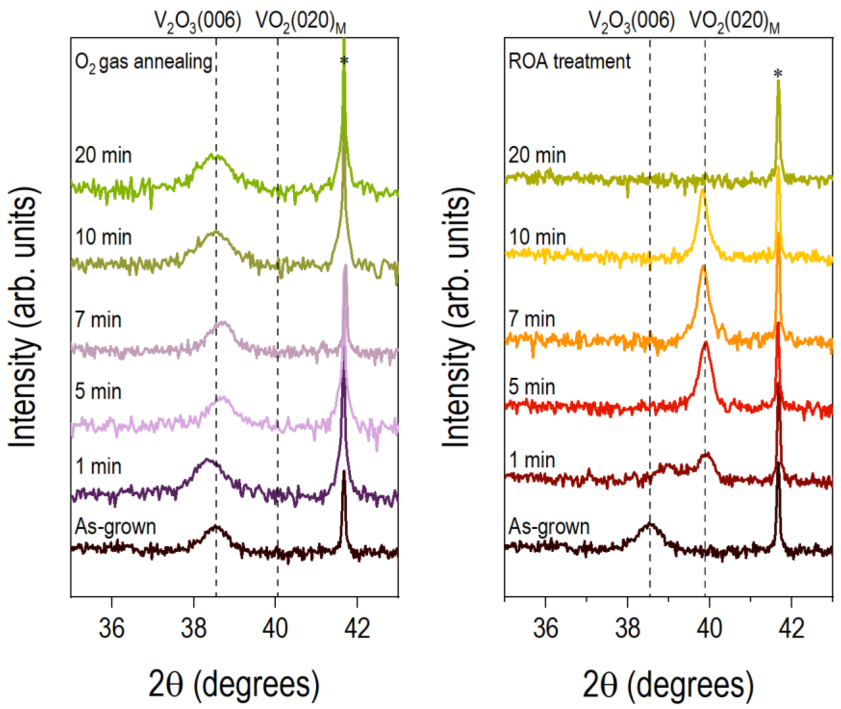

Figure S7. Comparison of 2θ/ω XRD patterns between post-annealed film under O_2 gas at 600 °C and ROA-treated film. Vertical dashed lines indicate the reference peak positions of V_2O_3(006) and VO_2(020).

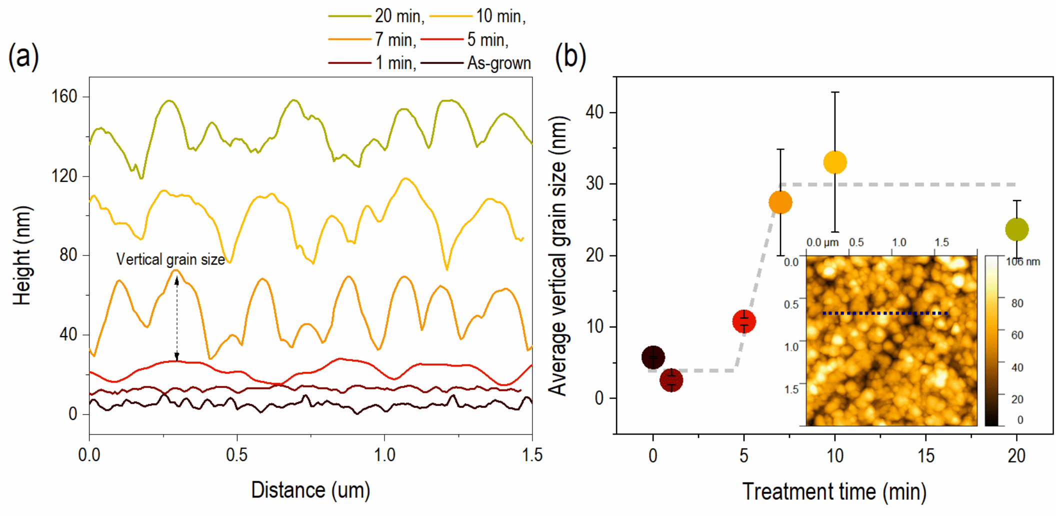

Figure S8. (a) Line profile and (b) average vertical grain size of the as-grown and ROA-treated vanadium oxide films. The inset in Fig. S8b shows the surface topology of 10 min-treated films. Average vertical grain size obtained by the difference between the highest and lowest points, as shown in Fig. S8a.

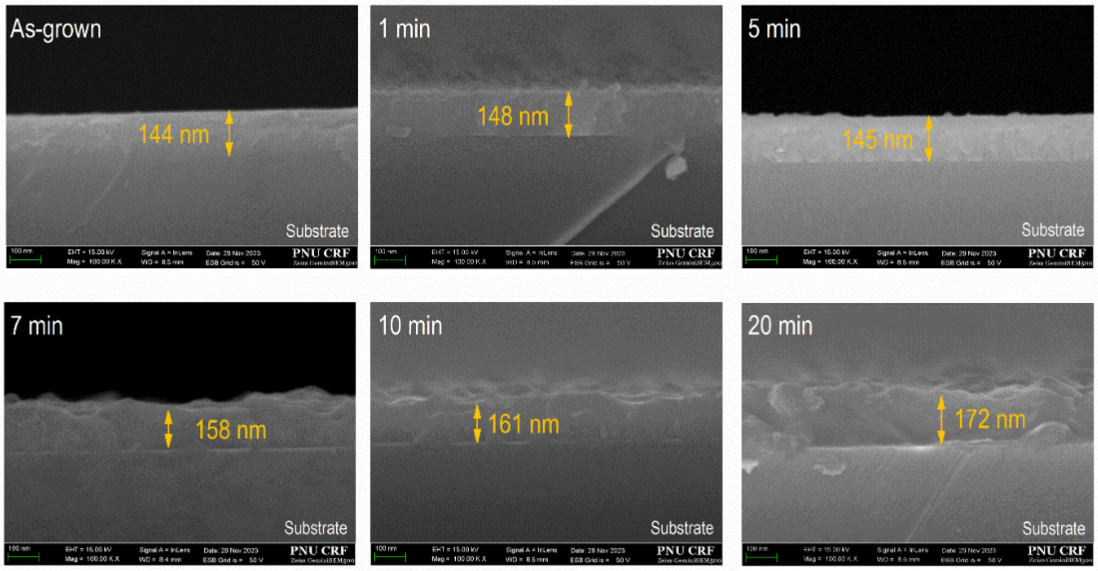

Figure S9. Cross-sectional FE-SEM image of the vanadium oxide films with increasing ROA time.

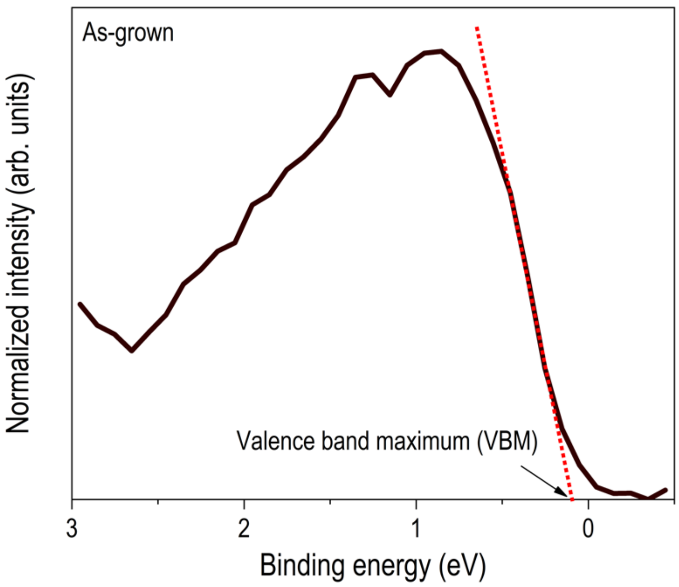

Figure S10. Extrapolation of valence band spectrum for the as-grown film.

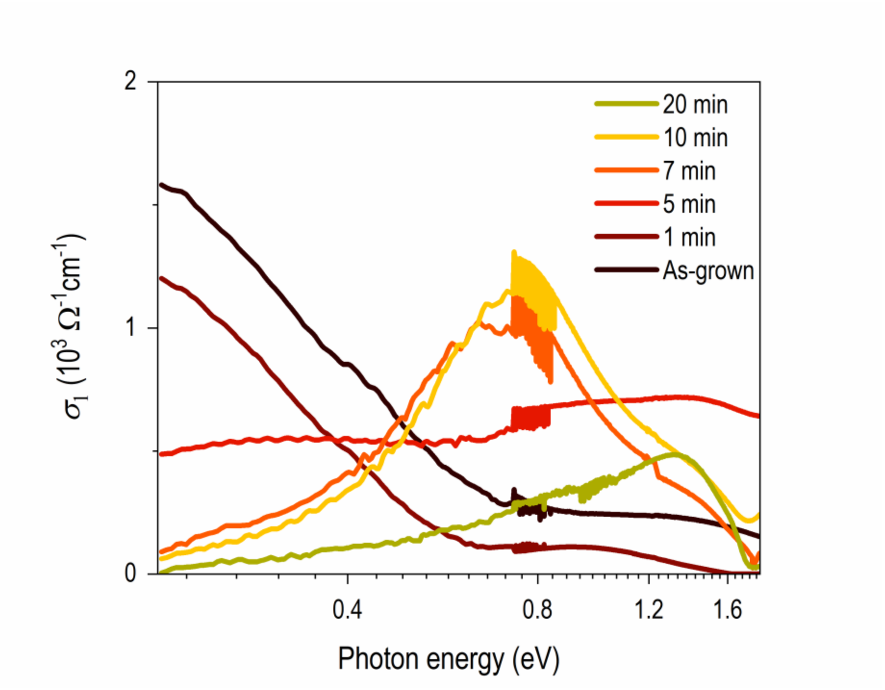

Figure S11. Real part of the optical conductivity (σ1) of the as-grown and ROA-treated films.

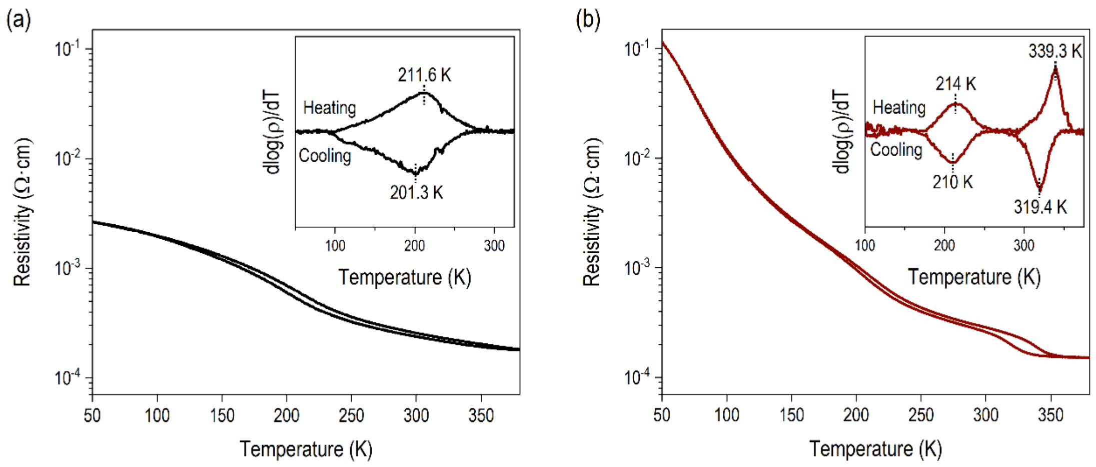

Figure S12. Temperature-dependent electrical resistivity of (a) as-grown and (b) 1 min ROA-treated vanadium oxide films. The insets show temperature-dependent differential log resistivity (dlog(ρ)/dT).

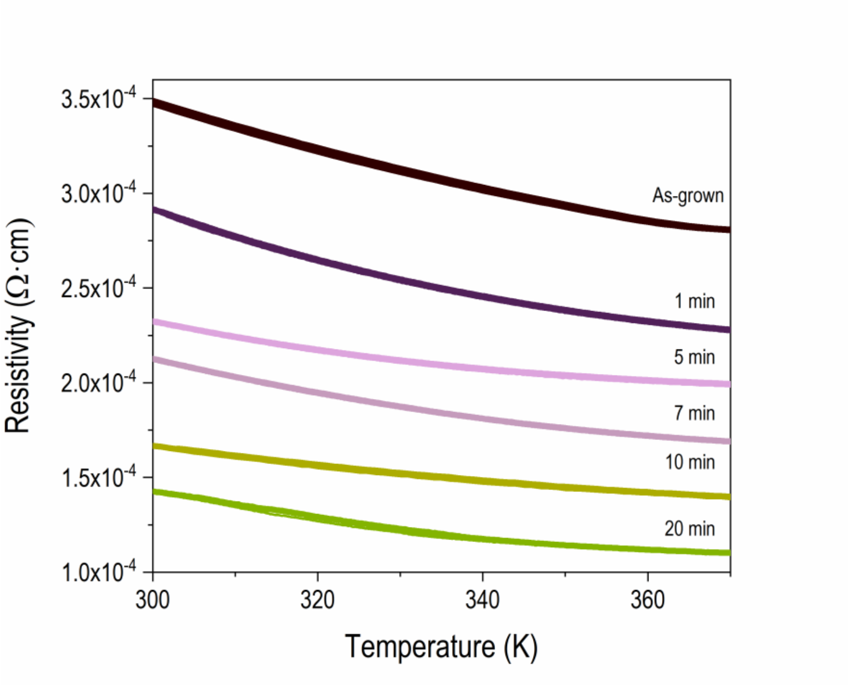

Figure S13. Temperature-dependent electrical resistivity of post-annealed films under O_2 gas at 600 ℃.
